# Overexpression of the translocon accessory protein YajC alleviates toxicity of the endogenous pore-forming toxin LdrA in *Escherichia coli*

**DOI:** 10.1371/journal.pone.0336059

**Published:** 2025-11-24

**Authors:** Yuto Nakamura, Yoshihiro Yamaguchi

**Affiliations:** Department of Biology, Graduate School of Science, Osaka Metropolitan University, Osaka, Japan; Federal University Dutse, NIGERIA

## Abstract

Almost all bacterial species possess toxin-antitoxin (TA) systems. In *Escherichia coli*, LdrA is a small membrane protein that functions as a pore-forming toxin. Expression of LdrA results in transient growth arrest, from which cells subsequently recover; however, the underlying recovery mechanism remained unknown. In the present study, we investigated the molecular basis for this recovery. Using a genetic screen, we identified YajC, an accessory protein of the SecDF-YajC translocon, as a key factor in counteracting of LdrA toxicity. We demonstrate that overexpression of YajC effectively alleviates LdrA toxicity without affecting its membrane localization or stability. This suppressive activity was specific for the Ldr toxin family, including LdrA and its homolog LdrD. Furthermore, our results indicate that YajC recognition of LdrA requires both its transmembrane and cytoplasmic domains. These findings reveal a novel function for YajC in counteracting a membrane-damaging toxin, distinct from its known role in the Sec-dependent protein translocation pathway.

## Introduction

The cell membrane is fundamental to bacterial life, serving as a selective barrier and the site for crucial processes including nutrient transport and energy generation via the proton motive force and ATP synthase [1,2]. Consequently, membrane integrity is a primary target for various antimicrobial agents and toxins [3]. Pathogenic bacteria utilize pore-forming toxins, such as α-hemolysin and leukocidins produced in *Staphylococcus aureus*, to disrupt host cell membranes and promote virulence [4–6]. Even non-pathogenic bacteria such as *Escherichia coli* encode proteins that can compromise their own membrane integrity. LdrA (Long Direct Repeat A) is a small, 35-amino acid, single-pass transmembrane protein that localizes to the cytoplasmic membrane [7]. Expression of LdrA results in the inhibition of ATP synthesis and ultimately leads to cell death, presumably through pore formation in the membrane. LdrA is a member of a homologous protein family in *E. coli*, which also includes LdrB, LdrC, and LdrD [8].

The *ldrA* gene constitutes a type I toxin-antitoxin (TA) system, where the expression of LdrA is negatively regulated at the translational level by *rdlA*-RNA, an antisense RNA encoded immediately upstream [8]. TA systems, typically composed of a stable toxin and a more labile antitoxin, are widespread regulatory modules found on the chromosomes and plasmids of most bacteria and archaea [9,10]. Under normal physiological conditions, the antitoxin effectively neutralizes the cognate toxin. However, under specific stress conditions, the antitoxin is degraded or inactivated, leading to the activation of toxin. These toxins can then inhibit diverse cellular processes, including DNA replication, mRNA stability, translation, or cell wall synthesis, often resulting in a bacteriostatic state or entry into dormancy [11–13].

During our initial characterization of LdrA, we made an intriguing observation: *E. coli* cells expressing this toxin exhibited only transient growth arrest, followed by a spontaneous recovery. This phenomenon strongly suggested the existence of an active cellular mechanism for counteracting LdrA toxicity. To identify the molecular components of this recovery mechanism, we performed a genetic screen. Here, we report the identification of YajC, an accessory protein of the SecDF-YajC translocon [14,15], as this neutralizing factor. Importantly, this neutralizing function of YajC appears to be distinct from its previously known role in protein translocation, thus revealing a novel function for YajC against an endogenous membrane-damaging toxin.

## Materials and methods

### Bacterial strains, plasmids, and growth conditions

*E. coli* strains MG1655, BL21(DE3), and DH10B were used in this study. For inducible expression, genes were cloned into pTC (pACYC184 derivative with tetracycline-inducible promoter; a kind gift from H. Nariya), pBAD24k (arabinose-inducible, Km^R^**)**, or pColdIV (IPTG-inducible, Amp^R^) [16]. The arabinose-inducible expression vector pBAD24k was constructed by replacing the ampicillin resistance gene of pBAD24 [17] with a kanamycin resistance cassette. Briefly, the pBAD24 backbone, excluding the *bla* gene, was PCR-amplified. A kanamycin resistance gene was also PCR-amplified from pET28a. These two fragments were then ligated using an In-Fusion Snap Assembly Master Mix (Takara Bio, Japan). Genes were amplified by PCR using primers (S1 Table. Used primers) and cloned into the respective vectors. To construct pColdIV-*yajC*-HA, the *yajC* gene was amplified and cloned into pColdIV, resulting in a C-terminal fusion with a hemagglutinin (HA) tag. LdrA-TisB chimeric constructs were generated using overlap extension PCR followed by In-Fusion Snap Assembly Master Mix into the pTC vector. All plasmid constructs were verified by Sanger sequencing. Bacteria were routinely grown in Luria-Bertani (LB) medium at 37°C with appropriate antibiotics (ampicillin 100 µg/ml, chloramphenicol 25 µg/ml and kanamycin 50 µg/ml). Inducers were added as specified: tetracycline (Tc) (0.1-0.25 µg/ml), L-arabinose (Ara) (0.2%), or isopropyl β-D-1-thiogalactopyranoside (IPTG) (0.1 mM).

### Genomic library construction and genetic screen

Genomic DNA from *E. coli* BW25113 was partially digested with Sau3AI to obtain fragments predominantly in the 2−8 kb range. These fragments were ligated into the BamHI site of pUC19 (Amp^R^). *E. coli* MG1655 cells harboring the pTC-*ldrA* plasmid (CmR) were transformed with the resulting library. Transformants were selected on LB agar plates containing ampicillin (100 µg/ml), chloramphenicol (25 µg/ml), and tetracycline (0.15 µg/ml) at 37°C for 18 h. Plasmids were isolated from resistant colonies, re-transformed into *E. coli* MG1655 harboring pTC-*ldrA* to confirm the LdrA suppression phenotype, and the inserts were identified by DNA sequencing from both ends using universal M13 forward and reverse primers. Site-directed deletion of the *yajC* gene from a complementing library clone (pUC19−7) was performed using the PrimeSTAR Mutagenesis Basal kit (Takara Bio) according to the manufacturer’s instructions, generating pUC19−7-∆*yajC*.

### Subcellular fractionation and western blotting

*E. coli* MG1655 expressing C-terminally FLAG-tagged LdrA (LdrA-FLAG) from the pTC vector, with or without co-expression of YajC from pColdIV, were harvested at indicated times after induction. Cell pellets were resuspended in lysis buffer [50 mM Tris-HCl (pH 8.0) and 150 mM NaCl]. Cells were lysed by sonication on ice. Unlysed cells and debris were removed by centrifugation (10,000 x *g*, 10 min, 4°C). The supernatant (whole cell lysate, W) was subjected to ultracentrifugation (100,000 x *g*, 1 h, 4°C) to separate the soluble fraction (S; cytoplasm and periplasm) from the membrane fraction (M). The membrane pellet was washed once with lysis buffer and resuspended in the same buffer. The protein from all fractions was acetone-precipitated and resuspended in 1 × SDS-PAGE sample buffer [50 mM Tris-HCl (pH 6.8), 10% (v/v) glycerol, 2% (w/v) SDS, 5% (v/v) β-mercaptoethanol, 0.01% (w/v) bromophenol blue]. The samples were analyzed by Bis-Tris PAGE followed by Western blotting using an anti-FLAG monoclonal antibody (Proteintech, Cat# 66008–4-Ig). For detection of LdrA-FLAG and the membrane fraction marker, a parallel blot prepared from the same set of samples was probed with a rabbit polyclonal anti-OmpA antibody (NBRP-E.coli at NIG). For detection of YajC-HA, an anti-HA polyclonal antibody (Proteintech, Cat# 51064–2-AP). The signals were developed using enhanced chemiluminescence substrate (Western BLoT Ultra Sensitive HRP Substrate, Takara Bio) and detected using a chemiluminescence imaging system (FUSION FX, Vilber Lourmat). Band intensities were quantified using Fiji (ImageJ). For quantification of LdrA-FLAG in the membrane fraction, the intensity of the LdrA-FLAG band was normalized to that of the OmpA band from the same lane. For quantification of YajC-HA, its band intensity was normalized to a non-specific cross-reactive band detected by the same antibody, which served as an internal loading control.

## Results

### LdrA expression causes transient growth arrest followed by recovery

**Fig 1:**
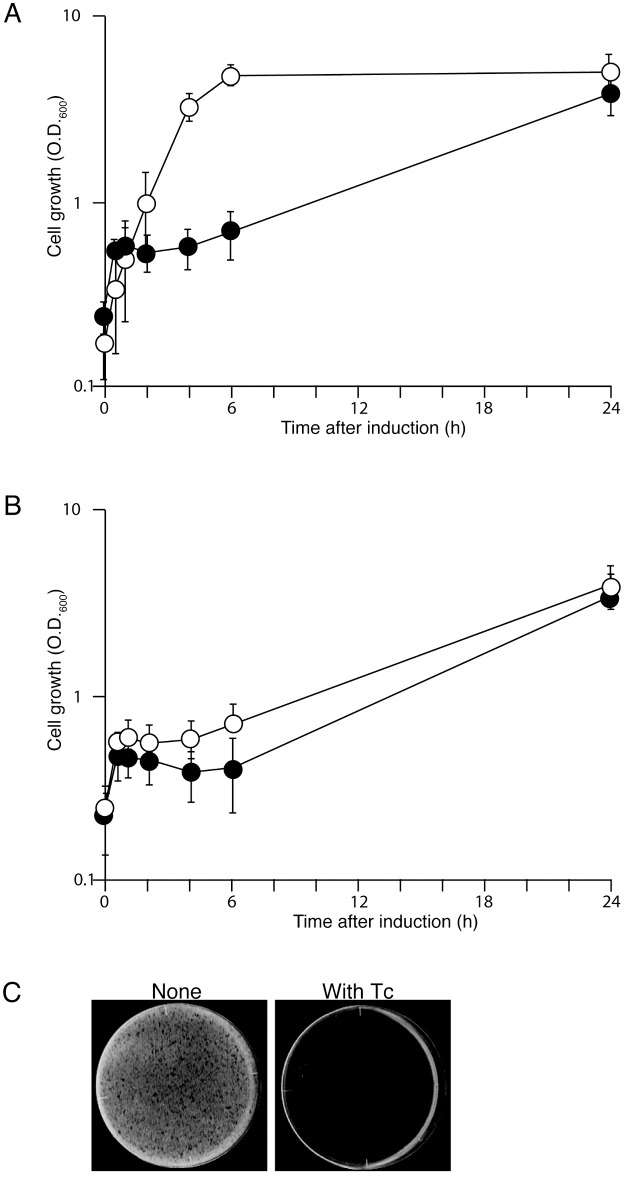
LdrA expression causes transient growth arrest and the FLAG tag does not affect its toxicity. **(A)** Growth curves of *E. coli* MG1655 harboring pTC (open circles) or pTC-*ldrA* (closed circles) after the addition of Tc. Cells were cultured in LB medium at 37°C. At an OD_600_ = 0.3, LdrA expression was induced by the addition of Tc (0.2 µg/ml). **(B)** Comparison of growth curves of cells expressing untagged LdrA (pTC-*ldrA*) or C-terminally FLAG-tagged LdrA (FLAG). **(C)** Plasmids recovered from regrown cells retain a functional *ldrA* gene. The pTC-*ldrA* plasmid was isolated from the regrown *E. coli* MG1655 culture shown in (A) 24 h after induction.. *E. coli* MG1655 cells were transformed with this recovered plasmid and spread on LB agar plates with or without Tc (0.25 µg/ml). Plates were incubated at 37°C for 16 h. Data in (A) and (B) are shown as mean ± SD from three independent experiments.

To investigate the physiological consequences of LdrA expression, *ldrA* was cloned under the control of a tetracycline (Tc)-inducible promoter in plasmid pTC-*ldrA*. Upon induction of LdrA in *E. coli* MG1655, a complete growth arrest was observed approximately 1 h after induction (Fig 1A). Remarkably, however, the culture spontaneously resumed growth around 5 h after induction, eventually reaching a cell density comparable to that of the uninduced control culture. All growth curve experiments were performed in biological triplicate, and the data are presented as mean ± standard deviation. To determine if this recovery was due to mutations inactivating the *ldrA* gene, we isolated the pTC-*ldrA* plasmid from cells that had recovered (24 h after LdrA induction). When this recovered plasmid was re-transformed into *E. coli*, the cells were unable to form colonies in the presence of Tc (Fig 1C). To confirm that the C-terminal FLAG tag used for detection did not interfere with LdrA’s toxic activity, we compared the growth of cells expressing tagged and untagged LdrA. The growth curves were nearly identical, indicating that LdrA-FLAG retains full toxic activity (Fig 1B, see also S1A Fig for spot assay). This result indicates that the *ldrA* gene on the recovered plasmid remained functionally active. We next investigated whether the observed recovery was due to the selection of stable, resistant mutants or a transient physiological adaptation. Cells that had recovered after 24 hours of LdrA induction were re-diluted into fresh medium and re-challenged with the inducer. These “pre-exposed” cells exhibited the same growth arrest kinetics as naïve cells that had never been exposed to LdrA (S1B Fig). Furthermore, we found that over 90% of the cells in the recovered population retained the pTC-*ldrA* plasmid, ruling out plasmid loss as a major mechanism of recovery (S1C Fig). Therefore, we conclude that the observed regrowth is not due to genetic inactivation of *ldrA*, but rather results from an active cellular process that appears to neutralize the LdrA toxicity.

### A genetic screen identifies YajC as an LdrA-neutralizing factor

**Fig 2:**
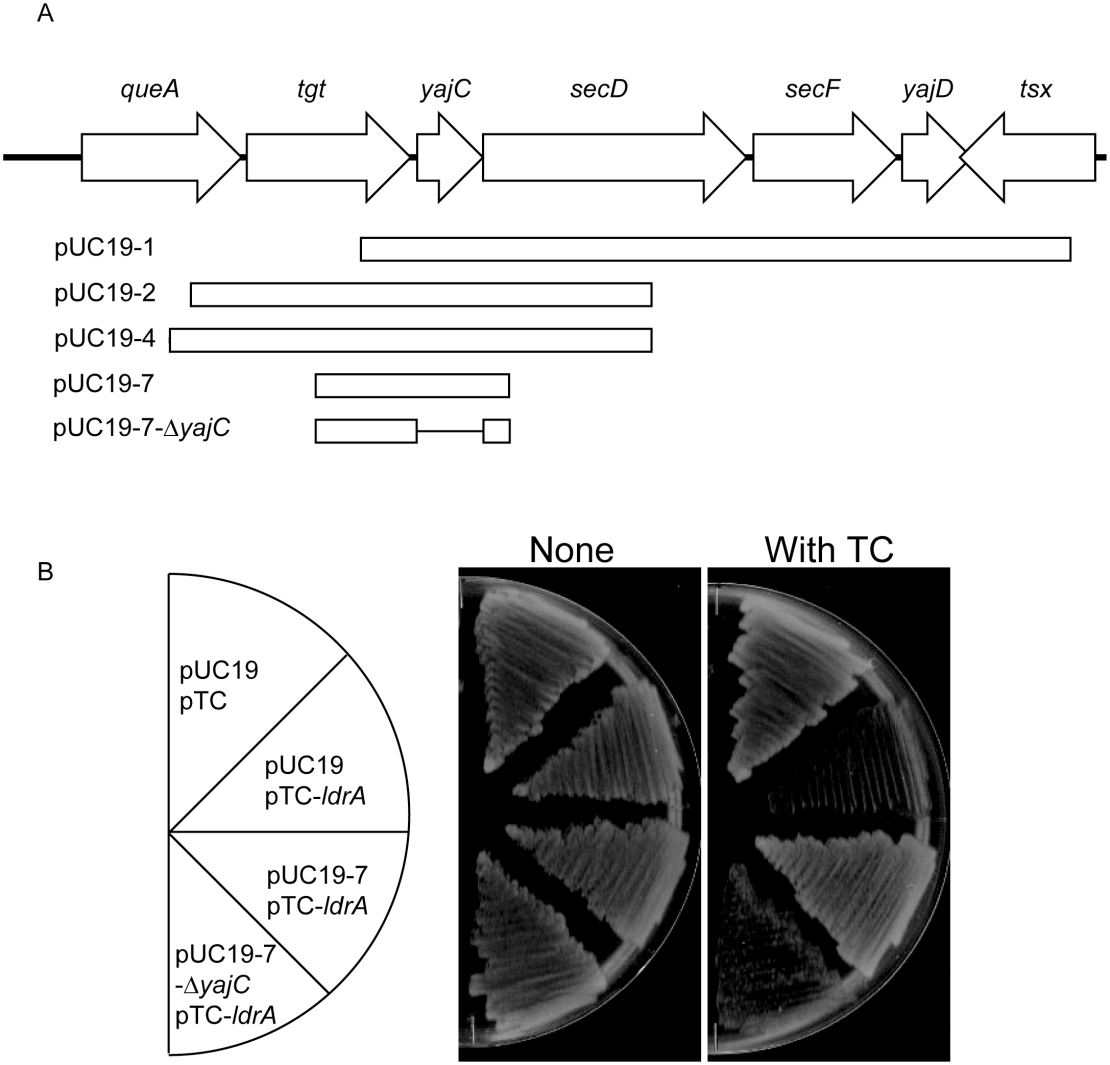
Genetic Screen Identifies YajC as an LdrA-Neutralizing Factor. **(A)** Schematic map of the *E. coli* chromosomal region containing *yajC*, identified in four independent complementing clones from the genomic library. Arrows indicate the orientation and extent of the genes in this region. The minimal overlapping region in the complementing clones is indicated. **(B)** Deletion of *yajC* abolishes LdrA resistance. *E. coli* MG1655 cells carrying pTC-*ldrA* were co-transformed with either empty pUC19 vector, the complementing library fragment pUC19−7, or pUC19−7 carrying a deletion of the *yajC* open reading frame (pUC19-7-Δ*yajC*). Cells were streaked on LB agar plates with or without Tc (0.25 µg/ml) and incubated at 37°C for 16 h.

To identify the host factor(s) responsible for LdrA neutralization, we performed a genetic screen. An *E. coli* genomic library constructed in pUC19 was transformed into MG1655 cells carrying pTC-*ldrA*. Transformants that grew on LB agar in the presence of Tc (0.15 µg/ml) were selected. Among approximately 26,000 transformants screened, five plasmids were isolated that reproducibly conferred resistance to LdrA. Sequence analysis revealed that one clone contained the *rob* gene, a known transcriptional activator that can upregulate efflux pumps [18], potentially reducing intracellular Tc concentration. Significantly, the remaining four independent clones all contained overlapping genomic DNA fragments that consistently encompassed the *yajC* gene region (Fig 2A). This finding strongly suggested that YajC is involved in neutralizing LdrA toxicity. To directly test this hypothesis, we specifically deleted the *yajC* open reading frame from one of the complementing library fragments (pUC19−7). This deletion completely abolished the ability of the fragment to suppress LdrA toxicity (Fig 2B). Thus, these results demonstrate that *yajC* within this fragment is required for the neutralization of LdrA.

### YajC overexpression is sufficient to alleviate LdrA toxicity

**Fig 3:**
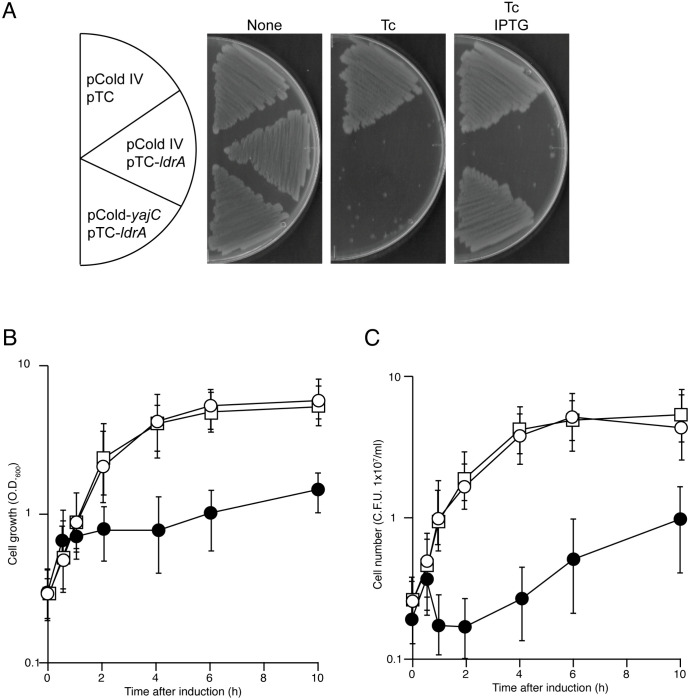
Overexpression of YajC is sufficient to alleviate LdrA-induced toxicity. **(A)** Effect of YajC overexpression on LdrA toxicity on solid medium. *E. coli* MG1655 cells harboring the indicated plasmid combinations were streaked onto LB agar plates containing 0.25 µg/ml Tc to induce LdrA and/or 0.1 mM IPTG to induce YajC. Plates were incubated at 37°C for 16 h. **(B and C)** Effect of YajC overexpression on LdrA toxicity in liquid culture. Cells harboring the plasmid combinations described in (A) were grown in LB medium at 37°C. YajC expression was pre-induced with IPTG (0.1 mM) for 15 min prior to the induction of LdrA expression with Tc (0.2 µg/ml) at an OD_600_ of 0.3. Growth curves were monitored by measuring OD_600_ **(B)**, and cell viability was monitored by determining colony-forming units (CFU/ml) **(C)**. Data in (B) and (C) are shown as mean ± SD from three independent experiments. Symbols: squares, pTC and pColdIV (vector controls); closed circles, pTC-*ldrA* and pColdIV; open circles, pTC-*ldrA* and pColdIV-*yajC*.

Next, we tested whether YajC overexpression alone was sufficient to counteract LdrA toxicity. We cloned *yajC* into an IPTG-inducible vector (pColdIV-*yajC*). *E. coli* carrying pTC-*ldrA* and pColdIV-*yajC* was able to form colonies on agar plates containing both Tc and IPTG. In contrast, growth of cells carrying pTC-*ldrA* and the empty pColdIV vector was completely inhibited (Fig 3A). We further examined the effect of YajC on LdrA in liquid culture. Co-expression of YajC allowed for continuous cell growth and maintained high viable cell counts (colony-forming units, CFU/ml) even after the induction of LdrA, closely mirroring the growth profile of control cells lacking LdrA (Fig 3B, 3C). Conversely, cells expressing LdrA without YajC co-expression exhibited rapid growth arrest and a significant decline in viability upon Tc addition. These results clearly demonstrate that YajC overexpression is sufficient to potently suppress LdrA function. Inducible expression of YajC-HA was confirmed by immunoblotting (S2A, B Fig).

### YajC had no effect on LdrA localization and stability

**Fig 4:**
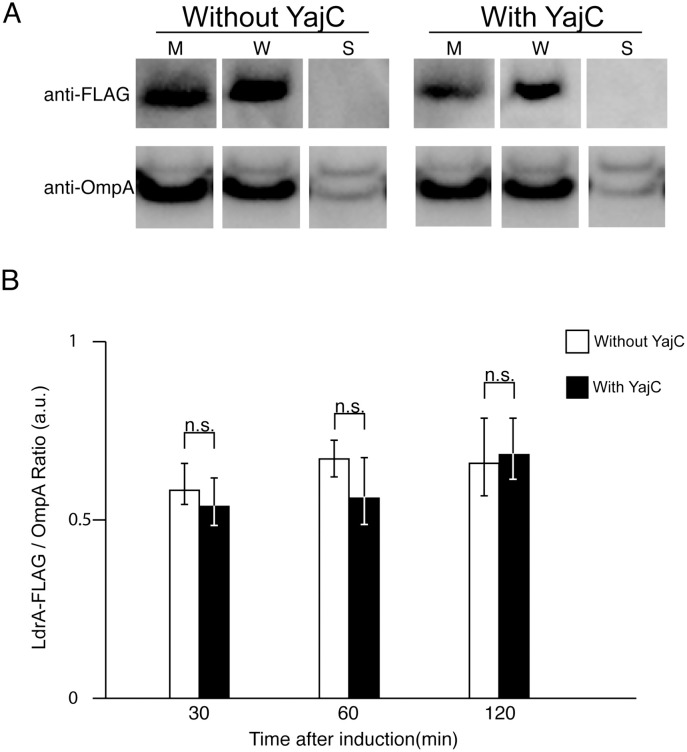
YajC co-expression does not alter the membrane localization or abundance of LdrA-FLAG. **(A)** Representative Western blot analysis of LdrA-FLAG subcellular localization. *E. coli* MG1655 cells co-expressing LdrA-FLAG with either an empty vector (-YajC) or with YajC (+YajC) were harvested 60 min after induction. Whole cell lysates **(W)**, soluble fractions **(S)**, and membrane fractions (M) were analyzed. The blot was probed with an anti-FLAG antibody (upper panel) to detect LdrA-FLAG, which localized predominantly to the membrane fraction. A parallel blot with identical samples was probed with an anti-OmpA antibody (lower panel), confirming the purity of the membrane fraction and serving as a loading control. **(B)** Quantification of membrane-associated LdrA-FLAG over time. Cells were harvested at 30, 60, and 120 min post-induction. The signal intensities of LdrA-FLAG in the membrane fractions were quantified from three independent experiments and normalized to the corresponding OmpA signal. Data are presented as mean ± SD. No significant differences were observed between cells with or without YajC co-expression at any time point (Student’s t-test; n.s., not significant).

To gain further insight into the mechanism by which YajC neutralizes LdrA, we examined whether YajC prevents the insertion of LdrA into the cell membrane or promotes its degradation. We expressed a C-terminally FLAG-tagged LdrA (LdrA-FLAG) with or without co-expressed YajC and analyzed subcellular localization of LdrA and levels over a 120-minute time course after induction. Subcellular fractions—whole cell lysate (W), soluble fraction (S), and membrane fraction (M)—were prepared. Western blot analysis showed that LdrA-FLAG was undetectable in the absence of the inducer tetracycline (Tc), confirming that its expression is tightly regulated (Fig 4A). Upon induction, LdrA-FLAG was detected predominantly in the membrane fraction, with no significant signal observed in the soluble fraction, regardless of whether YajC was co-expressed. The purity of the membrane fraction and uniform loading were confirmed with an antibody against the outer membrane protein OmpA, which was detected exclusively in the membrane fraction at comparable levels across all samples (Fig 4A, lower panel). The OmpA signal also served as a reliable loading control for the membrane fraction. LdrA-FLAG was detected predominantly in the membrane fraction, regardless of whether YajC was co-expressed (Fig 4A, upper panel). Quantitative analysis of the membrane fractions at 30, 60, and 120 minutes post-induction revealed that the amount of LdrA-FLAG did not significantly differ between cells with or without YajC overexpression at any time point (Fig 4B). Furthermore, the amount of LdrA localized in the membrane was stable over a 120 min after induction, with no evidence of accelerated degradation in the presence of YajC. These results suggest that YajC acts on LdrA after its insertion into the membrane, likely by interfering with its toxic function rather than by preventing its localization or inducing its rapid turnover. Full, uncropped blots corresponding to Fig 4A are provided in S3A, B Fig.

### YajC specifically neutralizes Ldr family toxins

**Fig 5:**
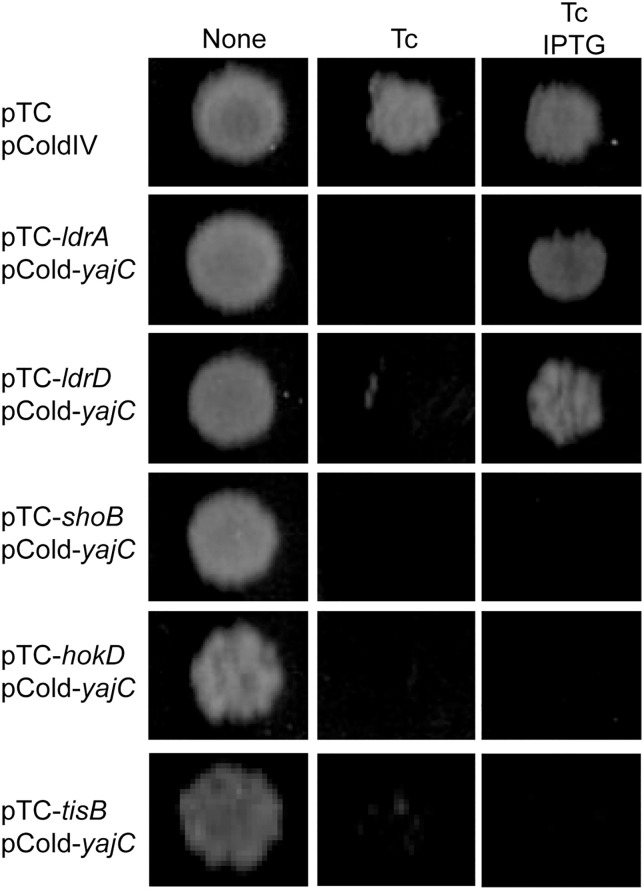
YajC Specifically Neutralizes Ldr Family Toxins. Spot dilution assay showing specificity of YajC-mediated neutralization. *E. coli* MG1655 cells harboring pBAD plasmids encoding LdrA, LdrD, TisB, ShoB, or HokD were co-transformed with pColdIV-*yajC*. Overnight cultures were serially diluted and spotted onto LB agar plates in the presence of Ara, IPTG or both. Plates were incubated at 37°C for 16 h.

To determine the specificity of neutralization mediated by YajC, we first tested if YajC could neutralize the toxicity of unrelated TA toxins, MqsR and RelE. MqsR and RelE are ribosome-independent and -dependent mRNA interferases, respectively, in *E. coli* [19,20]. YajC overexpression had no effect on their toxicity (S4 Fig). The data indicate that the neutralization effect of YajC against LdrA is not due to general mechanisms such as interference with inducer uptake or broad stress mitigation. We then tested the ability of YajC to counteract other known or putative pore-forming toxins, LdrD, ShoB, HokD and TisB [8, 21–23]. YajC overexpression successfully neutralized the toxicity of LdrD, a homolog of LdrA (74% amino acid identity). However, YajC had no effect on other pore-forming toxins TisB, ShoB, and HokD (Fig 5). Taken together, these findings demonstrate that YajC functions specifically to neutralize toxins belonging to the Ldr family, LdrA and its homolog LdrD.

### YajC recognition requires both transmembrane and cytoplasmic domains of LdrA

**Fig 6:**
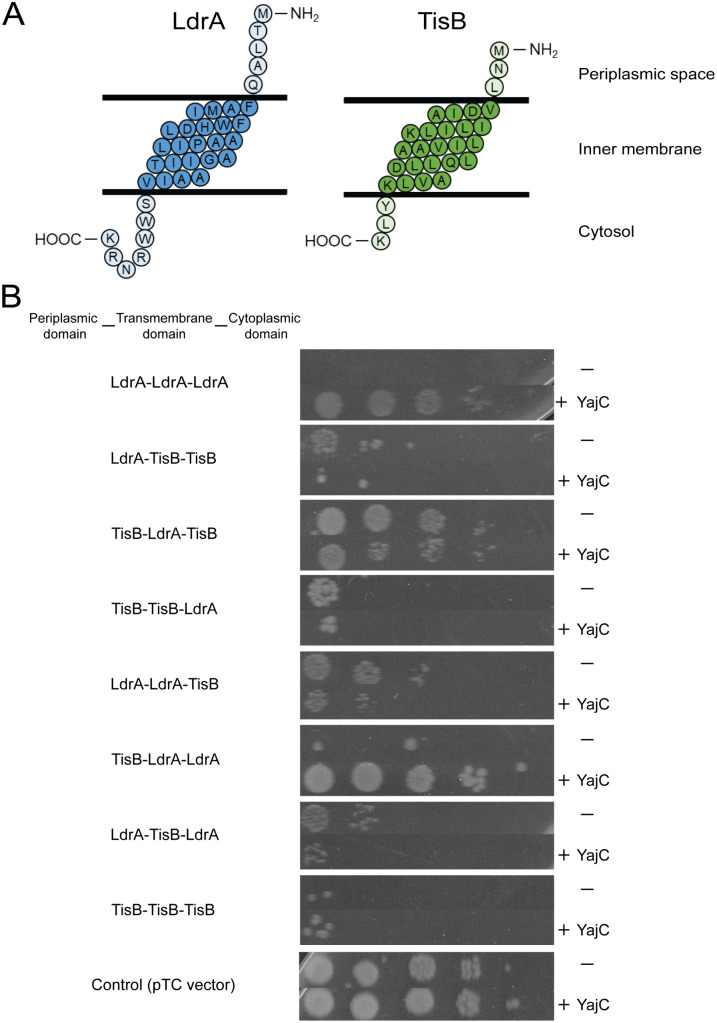
YajC recognition of LdrA requires both transmembrane and cytoplasmic domains. **(A)** Predicted membrane topology of LdrA and TisB proteins. **(B)** Effect of YajC co-expression on the toxicity of LdrA-TisB chimeric proteins. *E. coli* MG1655 cells harboring pTC plasmids encoding LdrA, TisB, or the indicated chimeric proteins were co-transformed with either an empty pColdIV vector or pColdIV-*yajC*. Overnight cultures were serially diluted and spotted onto LB agar plates containing 0.1 µg/ml Tc and 0.1 mM IPTG. Plates were incubated at 37°C for 16 h.

To map the determinants within LdrA required for YajC recognition and subsequent neutralization, we constructed a series of chimeric proteins between LdrA and TisB, another small pore-forming toxin that, as shown in Fig 5, is not neutralized by YajC. We systematically swapped their predicted periplasmic, transmembrane, and cytoplasmic domains based on topology predictions (Fig 6A). Expression of most of these chimeric proteins resulted in significant growth inhibition, suggesting that they retained pore-forming activity (Fig 6B, upper lane in each panel). We then tested the effect of YajC on the toxicity of these chimeras (Fig 6B, lower lane in each panel). YajC overexpression rescued cells from the toxicity caused by the chimera containing the LdrA transmembrane and LdrA cytoplasmic domains fused to the TisB periplasmic domain (TisB-LdrA-LdrA). However, YajC was unable to neutralize chimeras containing only the LdrA transmembrane domain (e.g., TisB-LdrA-TisB) or those lacking the LdrA cytoplasmic domain (e.g., LdrA-LdrA-TisB). These data therefore indicate that YajC recognizes a composite structural feature, involving both the transmembrane and cytoplasmic regions of LdrA, to mediate its neutralization.

## Discussion

In this study, we investigated the mechanism by which *E. coli* cells recover from the toxicity induced by the endogenous pore-forming toxin LdrA. Our results identify YajC as a key factor that effectively alleviates LdrA toxicity, and this YajC-mediated suppression may play a significant role in the observed recovery process. Our key findings show that YajC specifically neutralizes LdrA and its homolog LdrD. This suppression occurs after LdrA is inserted into the cell membrane, without promoting its degradation, and requires both the transmembrane and cytoplasmic domains of LdrA for recognition. Our identification of YajC, an accessory protein of the SecDF translocon [15,24,25] as a potent neutralizing factor of LdrA thus reveals an unexpected function for this protein, one that is distinct from its previously characterized role in the Sec-dependent protein translocation pathway.

The phenomenon of transient toxicity followed by recovery, as observed here with LdrA, shares similarities with behaviors described for other TA systems, which are often implicated in cellular transitions into and out of dormant or persistent states. For instance, a recovery mechanism has been well-characterized for the HokB toxin, another small membrane-acting pore-forming toxin in *E. coli*. HokB-induced dormancy can be reversed through the action of periplasmic redox enzymes (DsbA/DsbC) and eventual proteolytic degradation of HokB by the DegQ protease [13]. However, the YajC-mediated alleviation of LdrA toxicity appears to operate via a distinct mechanism. Our results provide no evidence for accelerated LdrA degradation in the presence of YajC (Fig 4), strongly suggesting that YajC employs a different strategy, possibly by interfering with the pore-forming activity of LdrA within the membrane, rather than promoting its degradation.

The identification of YajC as an LdrA neutralization factor was unanticipated, given its previously characterized role in protein translocation. This novel function prompted us to further consider its operational independence from the SecDF complex. Consistent with this idea, previous studies have suggested that YajC might possess functions independent of SecD and SecF, as YajC is significantly more abundant than SecD or SecF, exhibits a distinct phylogenetic distribution, and *yajC* deletion mutants display phenotypes distinguishable from those of *secDF* deletion mutants [25–27]. Our present findings are consistent with this notion; the observation that overexpression of *yajC* alone is sufficient to neutralize LdrA toxicity (Figs 2 and 3) strongly supports a model in which YajC functions independently of SecD and SecF in this protective role. Importantly, the absence of other consistently identified host factors in our genetic screen strongly suggests that YajC directly interacts with LdrA after its membrane insertion, inhibiting its activity.

Indeed, structural studies of YajC have revealed that one of its domains is structurally homologous to a domain of the multidrug efflux pump AcrB [28], suggesting that YajC possesses the structural capacity to interact with various membrane-embedded partners, potentially including small toxins like LdrA. The results obtained from the chimera analyses (Fig 6B) indicate that YajC recognition of LdrA indeed requires both its transmembrane and cytoplasmic domains. Taken together with our observation that YajC acts on LdrA post-membrane insertion without promoting its degradation (Fig 4), these findings provide strong evidence that YajC interferes with the function of membrane-embedded LdrA, likely through direct interaction involving specific recognition of these domains. However, our data, primarily based on genetic complementation and chimera analysis, do not definitively distinguish between direct inhibition (e.g., YajC binding to LdrA to block oligomerization or the pore formation) and other potential indirect mechanisms. Future biochemical studies using purified components would be essential to prove direct interaction between YajC and LdrA. While technically challenging due to the membrane protein nature of both components, these approaches are necessary for definitive mechanistic insight. Additionally, the physiological relevance of this YajC-mediated neutralization under native expression levels remains an important open question. Addressing these mechanistic and physiological questions represents significant future work that will provide deeper insights into the novel roles of YajC and the intricate defense strategies bacteria deploy against endogenous membrane stresses.

## Conclusions

In this study, we have unveiled a novel physiological role for the *E. coli* protein YajC, demonstrating its function as a potent and specific neutralizing factor against the endogenous pore-forming toxin LdrA. This YajC-mediated neutralization provides a molecular basis for the observed recovery of *E. coli* cells from LdrA-induced growth arrest. This finding significantly expands the known functional repertoire of YajC beyond protein transport, highlighting its involvement in a cellular defense mechanism against endogenous membrane-damaging agents. This work not only contributes to a better understanding of how bacteria cope with internal toxic stresses but also opens new avenues for investigating the multifaceted roles of proteins such as YajC in bacterial physiology and membrane homeostasis.

## Supporting information

S1 Table Used primers. List of primers used in this study (name and 5′ → 3′ sequence).
(DOCX)

S1 Fig Controls for LdrA toxicity and recovery experiments. (A) Spot assay comparing the toxicity of untagged LdrA and C-terminally FLAG-tagged LdrA. *E. coli* MG1655 cells harboring pTC-*ldrA* or pTC-*ldrA-*FLAG were serially diluted and spotted onto LB agar plates with or without 0.2 µg/ml Tc. (B) Recovery from LdrA toxicity is a phenotypic adaptation. *E. coli* MG1655 cells harboring pTC-*ldrA* that had recovered from a 24 h induction with Tc (“Pre-exposed cells”, open circles) were re-diluted into fresh medium and re-induced with Tc at an OD_600_ of 0.3. Their growth was compared to that of naïve cells induced for the first time (“Naïve cells”, closed circles). Data are shown as mean ± SD from three independent experiments. (C) High plasmid retention after recovery from LdrA toxicity. The percentage of cells retaining the pTC-*ldrA* plasmid was determined after 24 h of continuous induction. Retention was calculated by comparing colony-forming units (CFU) on non-selective and selective (chloramphenicol) LB agar plates. Data are shown as mean ± SD from three independent experiments.
(TIF)

S2 Fig Inducible overexpression of YajC-HA. (A) Western blot analysis of YajC-HA expression over time. *E. coli* MG1655 cells containing either an empty pColdIV vector (EV) or the pColdIV-*yajC-*HA plasmid were cultured to mid-log phase, and expression was induced with 0.1 mM IPTG at t = 0. Whole-cell lysates were collected at the indicated time points and analyzed by Western blotting using an anti-HA antibody. The asterisk denotes a non-specific cross-reactive protein band that served as an internal loading control. (B) Quantification of YajC-HA expression. The signal intensities of the YajC-HA band and the internal control band from the blot in (A) were quantified. The graph displays the ratio of the YajC-HA signal to the internal control signal. Data are representative of two independent experiments that yielded similar results.
(TIF)

S3 Fig Full, uncropped Western blot images corresponding to Fig 4A. Full-size, unprocessed images of the Western blots used for the analysis presented in Figure 4A. (A) The membrane was probed with an anti-FLAG antibody to detect LdrA-FLAG. (B) A parallel membrane, prepared with an identical set of samples, was probed with an anti-OmpA antibody. Lane order is as follows for both blots: 1, MW Marker; 2, -YajC, Membrane (30 min); 3, -YajC, Membrane (60 min); 4, -YajC, Membrane (120 min); 5, -YajC, Whole cell lysate (60 min); 6, -YajC, Soluble fraction (60 min); 7, + YajC, Membrane (30 min); 8, + YajC, Membrane (60 min); 9, + YajC, Membrane (120 min); 10, + YajC, Whole cell lysate (60 min); 11, + YajC, Soluble fraction (60 min); 12, Negative control (-Tc, 120 min), Membrane; 13, Negative control (-Tc, 120 min), Soluble fraction.
(TIF)

S4 Fig YajC has no effect on unrelated TA toxins. Growth assay of *E. coli* MG1655 harboring pBAD plasmids encoding indicated TA toxins, MqsR and RelE, co-transformed with empty pColdIV or pColdIV-*yajC*. Cells were spotted on LB agar plates containing 0.2% Ara with or without 0.1 mM IPTG.
(TIF)

## References

[1] Strahl H, Errington J. Bacterial Membranes: Structure, Domains, and Function. Annu Rev Microbiol. 2017;71:519–38. doi: 10.1146/annurev-micro-102215-095630 28697671

[2] Driessen AJM, Nouwen N. Protein translocation across the bacterial cytoplasmic membrane. Annu Rev Biochem. 2008;77:643–67. doi: 10.1146/annurev.biochem.77.061606.160747 18078384

[3] Epand RM, Walker C, Epand RF, Magarvey NA. Molecular mechanisms of membrane targeting antibiotics. Biochim Biophys Acta. 2016;1858(5):980–7. doi: 10.1016/j.bbamem.2015.10.018 26514603

[4] Bhakdi S, Tranum-Jensen J. Alpha-toxin of Staphylococcus aureus. Microbiol Rev. 1991;55(4):733–51. doi: 10.1128/mr.55.4.733-751.1991 1779933 PMC372845

[5] Alonzo F 3rd, Torres VJ. The bicomponent pore-forming leucocidins of Staphylococcus aureus. Microbiol Mol Biol Rev. 2014;78(2):199–230. doi: 10.1128/MMBR.00055-13 24847020 PMC4054254

[6] von Hoven G, Qin Q, Neukirch C, Husmann M, Hellmann N. Staphylococcus aureus α-toxin: small pore, large consequences. Biol Chem. 2019;400(10):1261–76. doi: 10.1515/hsz-2018-0472 30951494

[7] Yamaguchi Y, Tokunaga N, Inouye M, Phadtare S. Characterization of LdrA (long direct repeat A) protein of Escherichia coli. J Mol Microbiol Biotechnol. 2014;24(2):91–7. doi: 10.1159/000357949 24513967

[8] Kawano M, Oshima T, Kasai H, Mori H. Molecular characterization of long direct repeat (LDR) sequences expressing a stable mRNA encoding for a 35-amino-acid cell-killing peptide and a cis-encoded small antisense RNA in Escherichia coli. Mol Microbiol. 2002;45(2):333–49. doi: 10.1046/j.1365-2958.2002.03042.x 12123448

[9] Yamaguchi Y, Park J-H, Inouye M. Toxin-antitoxin systems in bacteria and archaea. Annu Rev Genet. 2011;45:61–79. doi: 10.1146/annurev-genet-110410-132412 22060041

[10] Jurėnas D, Fraikin N, Goormaghtigh F, Van Melderen L. Biology and evolution of bacterial toxin-antitoxin systems. Nat Rev Microbiol. 2022;20(6):335–50. doi: 10.1038/s41579-021-00661-1 34975154

[11] Dörr T, Vulić M, Lewis K. Ciprofloxacin causes persister formation by inducing the TisB toxin in Escherichia coli. PLoS Biol. 2010;8(2):e1000317. doi: 10.1371/journal.pbio.1000317 20186264 PMC2826370

[12] Edelmann D, Berghoff BA. Type I toxin-dependent generation of superoxide affects the persister life cycle of Escherichia coli. Sci Rep. 2019;9(1). doi: 10.1038/s41598-019-50668-1 PMC6776643 31582786

[13] Wilmaerts D, Dewachter L, De Loose P-J, Bollen C, Verstraeten N, Michiels J. HokB Monomerization and Membrane Repolarization Control Persister Awakening. Mol Cell. 2019;75(5):1031-1042.e4. doi: 10.1016/j.molcel.2019.06.015 31327636

[14] Oswald J, Njenga R, Natriashvili A, Sarmah P, Koch H-G. The Dynamic SecYEG Translocon. Front Mol Biosci. 2021;8:664241. doi: 10.3389/fmolb.2021.664241 33937339 PMC8082313

[15] du Plessis DJF, Nouwen N, Driessen AJM. The Sec translocase. Biochim Biophys Acta. 2011;1808(3):851–65. doi: 10.1016/j.bbamem.2010.08.016 20801097

[16] Qing G, Ma L-C, Khorchid A, Swapna GVT, Mal TK, Takayama MM, et al. Cold-shock induced high-yield protein production in Escherichia coli. Nat Biotechnol. 2004;22(7):877–82. doi: 10.1038/nbt984 15195104

[17] Guzman LM, Belin D, Carson MJ, Beckwith J. Tight regulation, modulation, and high-level expression by vectors containing the arabinose PBAD promoter. J Bacteriol. 1995;177(14):4121–30. doi: 10.1128/jb.177.14.4121-4130.1995 7608087 PMC177145

[18] Martin RG, Rosner JL. Analysis of microarray data for the marA, soxS, and rob regulons of Escherichia coli. Methods Enzymol. 2003;370:278–80. doi: 10.1016/S0076-6879(03)70024-X 14712652

[19] Yamaguchi Y, Park J-H, Inouye M. MqsR, a crucial regulator for quorum sensing and biofilm formation, is a GCU-specific mRNA interferase in Escherichia coli. J Biol Chem. 2009;284(42):28746–53. doi: 10.1074/jbc.M109.032904 19690171 PMC2781420

[20] Pedersen K, Zavialov AV, Pavlov MY, Elf J, Gerdes K, Ehrenberg M. The bacterial toxin RelE displays codon-specific cleavage of mRNAs in the ribosomal A site. Cell. 2003;112(1):131–40. doi: 10.1016/s0092-8674(02)01248-5 12526800

[21] Fozo EM, Kawano M, Fontaine F, Kaya Y, Mendieta KS, Jones KL, et al. Repression of small toxic protein synthesis by the Sib and OhsC small RNAs. Mol Microbiol. 2008;70(5):1076–93. doi: 10.1111/j.1365-2958.2008.06394.x 18710431 PMC2597788

[22] Pedersen K, Gerdes K. Multiple hok genes on the chromosome of Escherichia coli. Mol Microbiol. 1999;32(5):1090–102. doi: 10.1046/j.1365-2958.1999.01431.x 10361310

[23] Vogel J, Argaman L, Wagner EGH, Altuvia S. The small RNA IstR inhibits synthesis of an SOS-induced toxic peptide. Curr Biol. 2004;14(24):2271–6. doi: 10.1016/j.cub.2004.12.003 15620655

[24] Nouwen N, Driessen AJM. SecDFyajC forms a heterotetrameric complex with YidC. Mol Microbiol. 2002;44(5):1397–405. doi: 10.1046/j.1365-2958.2002.02972.x 12068816

[25] Pogliano KJ, Beckwith J. Genetic and molecular characterization of the Escherichia coli secD operon and its products. J Bacteriol. 1994;176(3):804–14. doi: 10.1128/jb.176.3.804-814.1994 7507921 PMC205118

[26] Taura T, Akiyama Y, Ito K. Genetic analysis of SecY: additional export-defective mutations and factors affecting their phenotypes. Mol Gen Genet. 1994;243(3):261–9. doi: 10.1007/BF00301061 8190079

[27] Hand NJ, Klein R, Laskewitz A, Pohlschröder M. Archaeal and bacterial SecD and SecF homologs exhibit striking structural and functional conservation. J Bacteriol. 2006;188(4):1251–9. doi: 10.1128/JB.188.4.1251-1259.2006 16452406 PMC1367261

[28] Törnroth-Horsefield S, Gourdon P, Horsefield R, Brive L, Yamamoto N, Mori H, et al. Crystal structure of AcrB in complex with a single transmembrane subunit reveals another twist. Structure. 2007;15(12):1663–73. doi: 10.1016/j.str.2007.09.023 18073115

