# Supplementary material for: Overexpression of the translocon accessory protein YajC alleviates toxicity of the endogenous pore-forming toxin LdrA in Escherichia coli
Source: PLoS One. 2025 Nov 24;20(11):e0336059. doi: 10.1371/journal.pone.0336059 (PMC12643268; doi:10.1371/journal.pone.0336059)
Supplement: S1 Table — List of primers used in this study (name and 5′ → 3′ sequence). (DOCX) [file pone.0336059.s001.docx]

S1 Table. Used primers

| Primer name | Primer sequence (5’→3’) |
| --- | --- |
| ldrA for pTC Fw | GAGAAAAGTCATATGACGCTCGCGCAGTT |
| ldrA for pTC Rv | GAGGGTACCGAGCTCTTACTTCCGGTTACGCCA |
| yajC Fw | GAGGTAATACCATATGAGCTTTTTTATTTCTGATGC |
| yajC Rv | TCGAGGGTACCGAGCTCTTACAGCGCCTTCATGGTGCCTTT |
| pUC19-7 w/o yajC Fw | TTAAAATTTTTCCTAAGGGAATTGC |
| pUC19-7 w/o yajC Rv | AGGGAAAAATTTTAATAAATTTCCCTCATTATTAATATTAATCA |
| ldrA-FLAG Fw | GACTACAAGGATGATGATGACAAATAAGAGCTCGGTACCCTCGA |
| ldrA FLAG Rv | TTTGTCATCATCATCCTTGTAGTCCTTCCGGTTACGCCACC |
| M13 Fw | GTAAAACGACGGCCAGT |
| M13 Rv | CAGGAAACAGCTATGAC |
| ldrD for pTC Fw | GAGAAAAGTCATATGACGTTCGCAGAGC |
| ldrD for pTC Rv | GAGGGTACCGAGCTCTTACTTCCGCTTGTTCA |
| shoB for pTC Fw | GAGAAAAGTCATATGACTGATTGCCGAT |
| shoB for pTC Rv | GAGGGTACCGAGCTCTTATAAGAACAGAAGGA |
| hokD for pTC Fw | GAGAAAAGTCATATGAAGCAGCAAAAGG |
| hokD for pTC Rv | GAGGGTACCGAGCTCTTACTCCTCAGGTTCGTA |
| tisB for pTC Fw | GAGAAAAGTCATATGAAGCAGCAAAAGG |
| tisB for pTC Rv | GAGGGTACCGAGCTCTTACTCCTCAGGTTCGTA |
| ldrA-tisB-ldrA Fw | GAGGTAATACCATATGACGCTCGCGCAGGTGGATATCGCCATTCTTATCC |
| ldrA-tisB-ldrA Rv | CGAGGGTACCGAGCTCTTACTTCCGGTTACGCCACCAGCTTTTCAGAACAGCATCAAGCA |
| tisB-ldrA-tisB Fw | GAGGTAATACCATATGAACCTGTTTGCCATGATTTTCTGGCACGACCTGGCAGCACCGATCCTGGCGGGAA |
| tisB-ldrA-tisB Rv | CGAGGGTACCGAGCTCTTACTTCAGGTAGACAATCGCTGCGGTAATAATTCCCGCCAGGATCGGTGCTGC |
| ldrA-tisB-ldrA Fw  ldrA-tisB-ldrA Rv  ldrA-ldrA-tisB Fw  ldrA-ldrA-tisB Rv  tisB-ldrA-ldrA Fw  tisB-ldrA-ldrA Rv  ldrA-tisB-tisB Fw  ldrA-tisB-tisB Rv  tisB-tisB-ldrA Fw  tisB-tisB -ldrA Rv | GAGGTAATACCATATGACGCTCGCGCAGGTGGATATCGCCATTCTTATGC  CGAGGGTACCGAGCTCTTACTTCCGGTTACGCCACCAGCTTTTCAGAACAGCATCAA  TACCTGAAGTAAGAGCTCGGTACCCTCG  CTCTTACTTCAGGTAGACAATCGCTGCGGTAATAATT  CAGGTTCATATGGTATTACCTCTTAACTGATAG  ATACCATATGAACCTGTTTGCCATGATTTTCTGGCAC  AATTACCTGAAGTAAGAGCTCGGTACCCTCG  TTACTTCAGGTATTTCAGAACAGCATCAAGCAG  GGTGGCGTAACCGGAAGTAAGAGCTCGGTACCCTCG  TCCGGTTACGCCACCAGCTTTTCAGAACAGCATCAAGCAG |
