# Supplementary figures and images for: Overexpression of the translocon accessory protein YajC alleviates toxicity of the endogenous pore-forming toxin LdrA in Escherichia coli
Source: PLoS One. 2025 Nov 24;20(11):e0336059. doi: 10.1371/journal.pone.0336059 (PMC12643268; doi:10.1371/journal.pone.0336059)

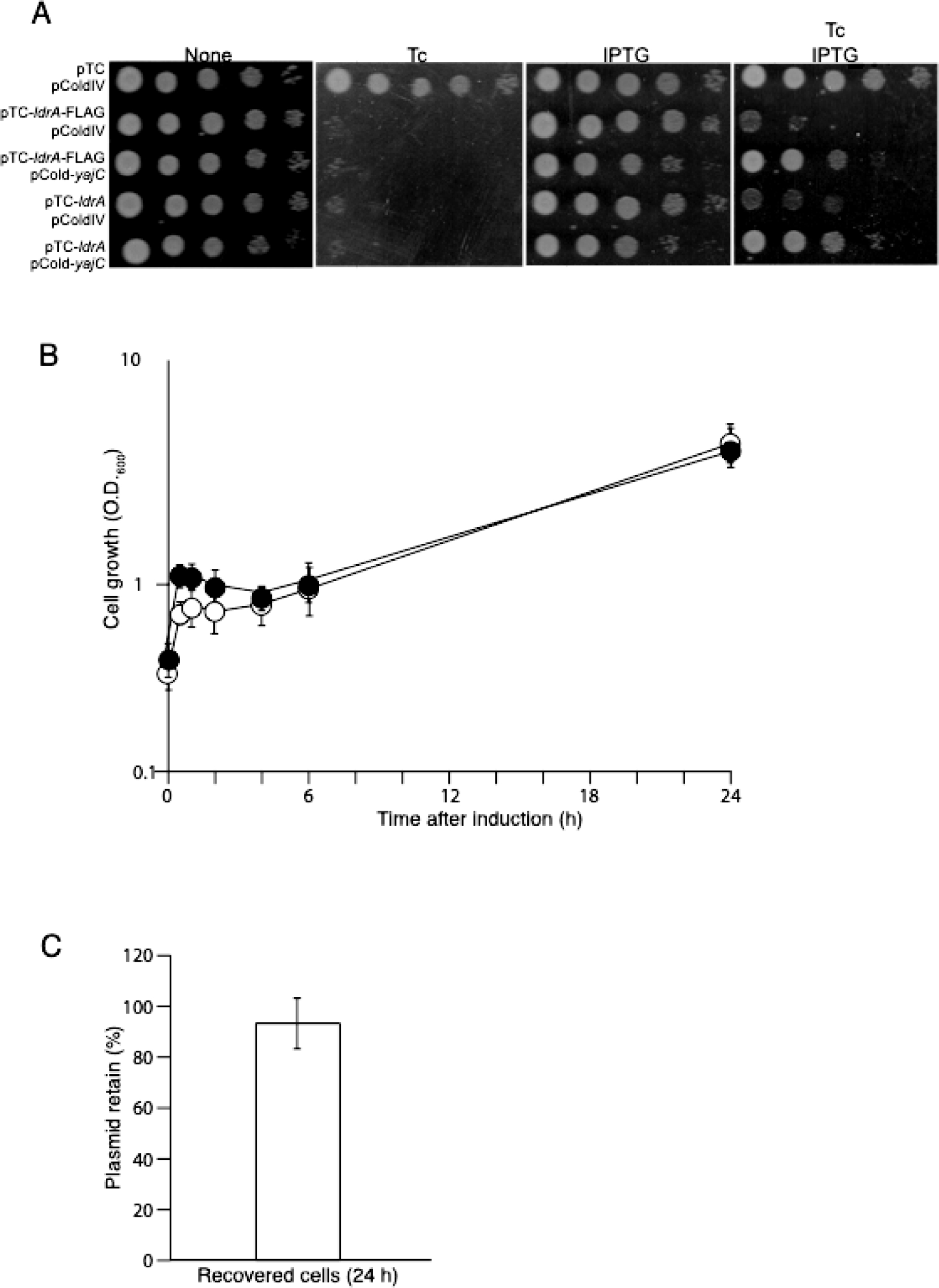

Supplement: S1 Fig — (A) Spot assay comparing the toxicity of untagged LdrA and C-terminally FLAG-tagged LdrA. E. coli MG1655 cells harboring pTC-ldrA or pTC-ldrA-FLAG were serially diluted and spotted onto LB agar plates with or without 0.2 µg/ml Tc. (B) Recovery from LdrA toxicity is a phenotypic adaptation. E. coli MG1655 cells harboring pTC-ldrA that had recovered from a 24 h induction with Tc (“Pre-exposed cells”, open circles) were re-diluted into fresh medium and re-induced with Tc at an OD600 of 0.3. Their growth was compared to that of naïve cells induced for the first time (“Naïve cells”, closed circles). Data are shown as mean ± SD from three independent experiments. (C) High plasmid retention after recovery from LdrA toxicity. The percentage of cells retaining the pTC-ldrA plasmid was determined after 24 h of continuous induction. Retention was calculated by comparing colony-forming units (CFU) on non-selective and selective (chloramphenicol) LB agar plates. Data are shown as mean ± SD from three independent experiments. (TIF) [file pone.0336059.s002.tif]

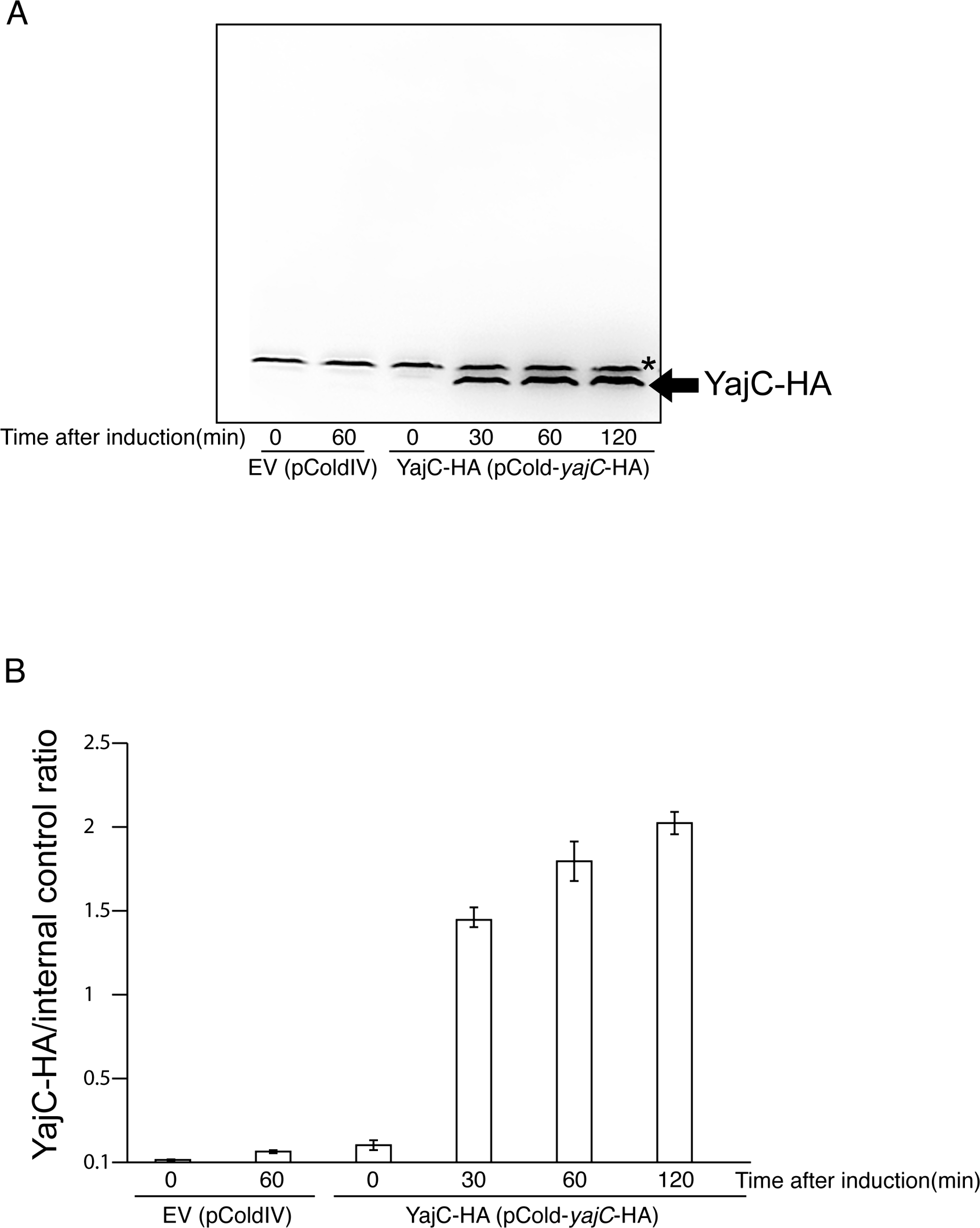

Supplement: S2 Fig — (A) Western blot analysis of YajC-HA expression over time. E. coli MG1655 cells containing either an empty pColdIV vector (EV) or the pColdIV-yajC-HA plasmid were cultured to mid-log phase, and expression was induced with 0.1 mM IPTG at t = 0. Whole-cell lysates were collected at the indicated time points and analyzed by Western blotting using an anti-HA antibody. The asterisk denotes a non-specific cross-reactive protein band that served as an internal loading control. (B) Quantification of YajC-HA expression. The signal intensities of the YajC-HA band and the internal control band from the blot in (A) were quantified. The graph displays the ratio of the YajC-HA signal to the internal control signal. Data are representative of two independent experiments that yielded similar results. (TIF) [file pone.0336059.s003.tif]

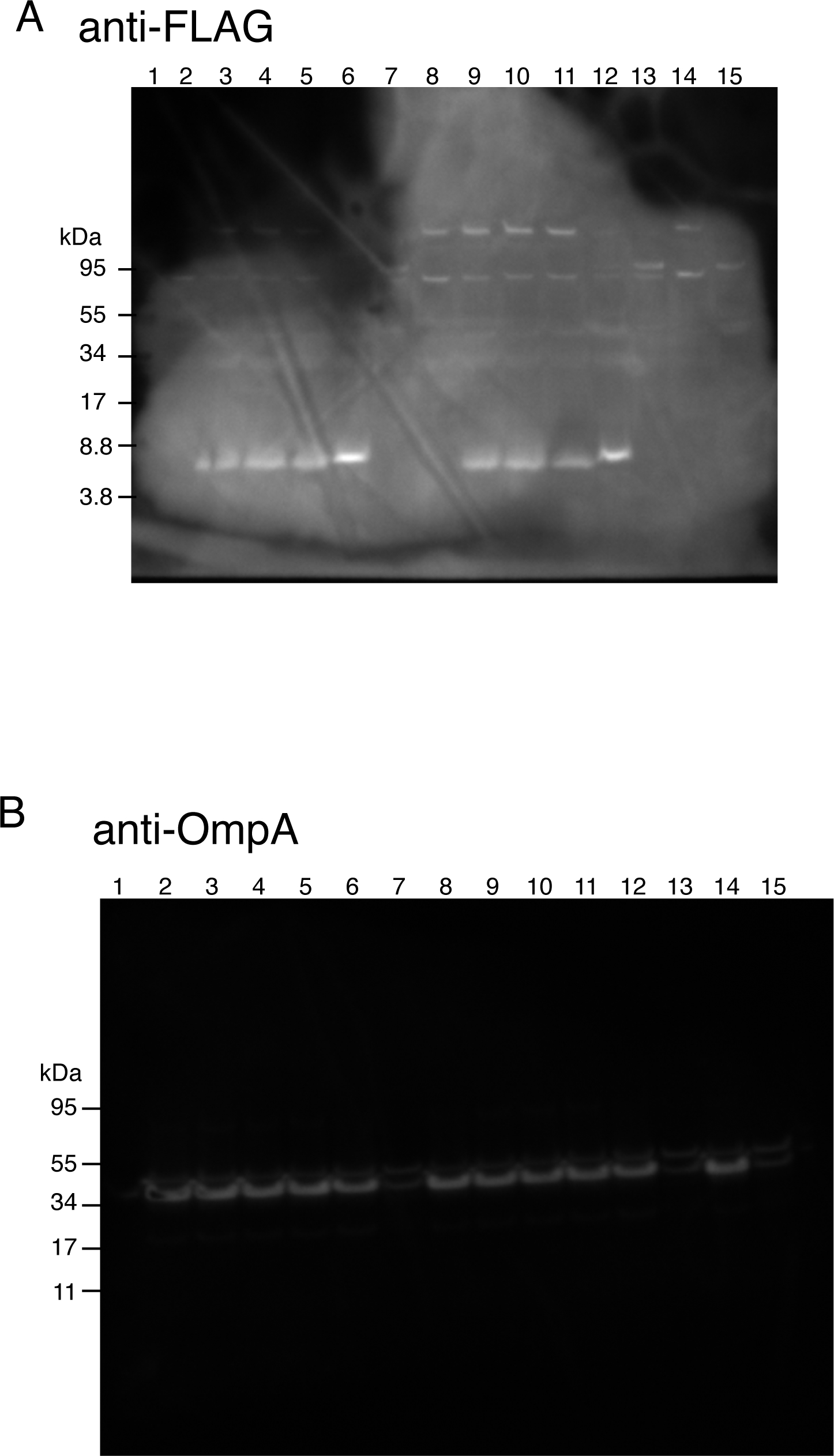

Supplement: S3 Fig — Full-size, unprocessed images of the Western blots used for the analysis presented in Figure 4A. (A) The membrane was probed with an anti-FLAG antibody to detect LdrA-FLAG. (B) A parallel membrane, prepared with an identical set of samples, was probed with an anti-OmpA antibody. Lane order is as follows for both blots: 1, MW Marker; 2, -YajC, Membrane (30 min); 3, -YajC, Membrane (60 min); 4, -YajC, Membrane (120 min); 5, -YajC, Whole cell lysate (60 min); 6, -YajC, Soluble fraction (60 min); 7, + YajC, Membrane (30 min); 8, + YajC, Membrane (60 min); 9, + YajC, Membrane (120 min); 10, + YajC, Whole cell lysate (60 min); 11, + YajC, Soluble fraction (60 min); 12, Negative control (-Tc, 120 min), Membrane; 13, Negative control (-Tc, 120 min), Soluble fraction. (TIF) [file pone.0336059.s004.tif]

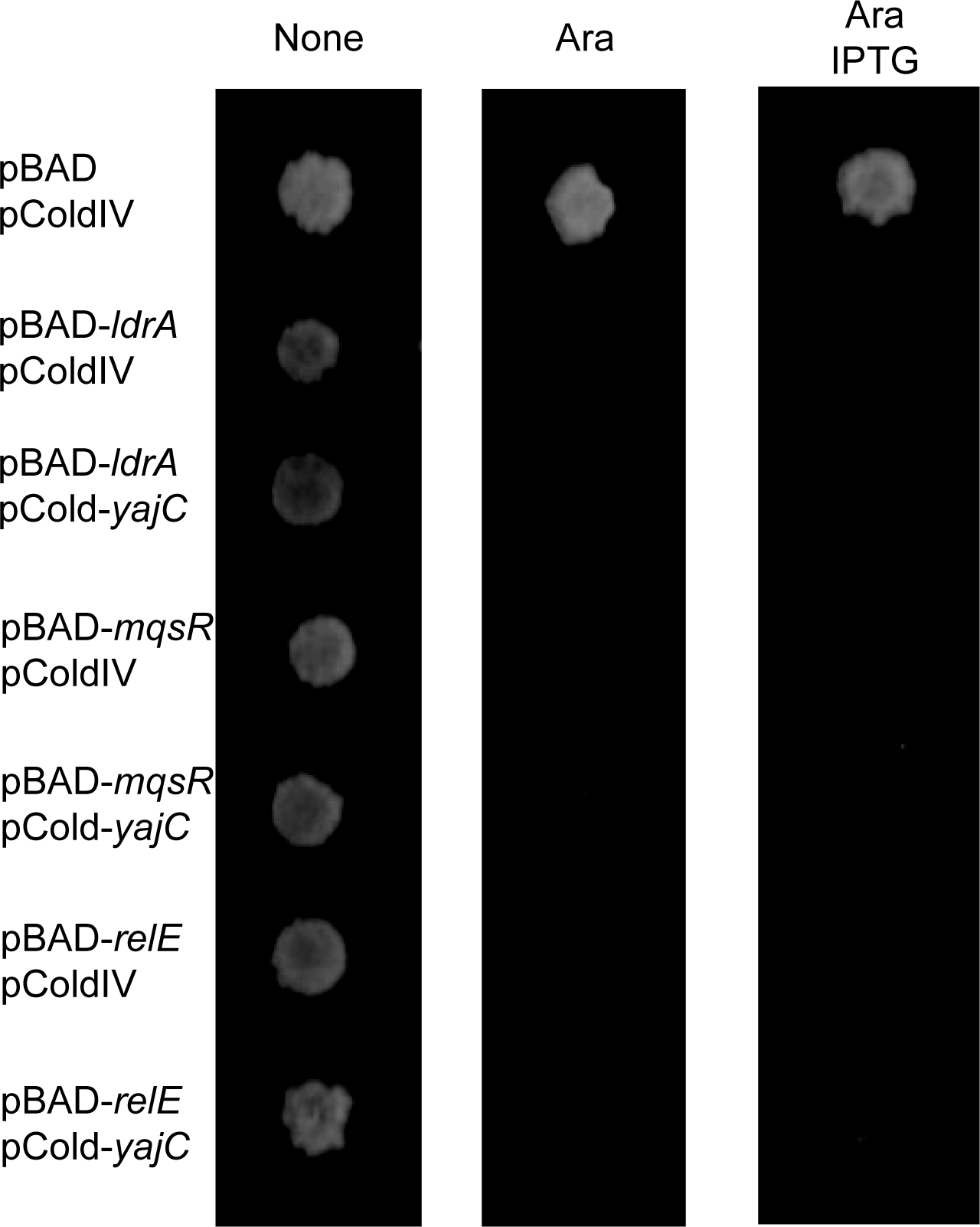

Supplement: S4 Fig — Growth assay of E. coli MG1655 harboring pBAD plasmids encoding indicated TA toxins, MqsR and RelE, co-transformed with empty pColdIV or pColdIV-yajC. Cells were spotted on LB agar plates containing 0.2% Ara with or without 0.1 mM IPTG. (TIF) [file pone.0336059.s005.tif]
